# Neuromuscular Blockade Correlates with Hormones and Body Composition in Acromegaly

**DOI:** 10.1155/2020/2912839

**Published:** 2020-12-09

**Authors:** Yu Zhang, Xiaopeng Guo, Gang Tan, Mengyun Zhao, Yuguang Huang, Wei Chen, Xiaodong Shi, Lijian Pei, Bing Xing

**Affiliations:** ^1^Department of Anesthesiology, Peking Union Medical College Hospital, Chinese Academy of Medical Sciences & Peking Union Medical College, No. 1 Shuaifuyuan, Dongcheng, Beijing 100730, China; ^2^Department of Neurosurgery, Peking Union Medical College Hospital, Chinese Academy of Medical Sciences & Peking Union Medical College, No. 1 Shuaifuyuan, Dongcheng, Beijing 100730, China; ^3^China Pituitary Disease Registry Center, China Pituitary Adenoma Specialist Council, No. 1 Shuaifuyuan, Dongcheng, Beijing 100730, China; ^4^Department of Parenteral & Enteral Nutrition, Peking Union Medical College Hospital, Chinese Academy of Medical Sciences & Peking Union Medical College, No. 1 Shuaifuyuan, Dongcheng, Beijing 100730, China; ^5^Outcomes Research Consortium, 9500 Euclid Ave, Cleveland 44195, Ohio, USA

## Abstract

Tumor resection is the first-line therapy for acromegaly patients. In some cases, unsatisfactory intraoperative neuromuscular blockades (NMBs) lead to failed operations. The purpose of this study was to investigate and quantify the NMB status of acromegaly patients and explore the relationship between NMB status and hormone levels and body composition. Twenty patients with untreated acromegaly and seventeen patients with nonfunctioning pituitary adenomas as controls were enrolled in this study. NMB was assessed using the train-of-four (TOF) technique with TOF-Watch® SX. The onset time of NMB, deep neuromuscular blockade duration (DNMBD), and clinical neuromuscular blockade duration (CNMBD) were monitored. We found a significantly longer onset time (110.25 ± 54.90 vs. 75.00 ± 27.56, s, *p*=0.017), shorter DNMBD (21.99 ± 5.67 vs. 34.96 ± 11.04, min, *p* < 0.001), and shorter CNMBD (33.26 ± 8.09 vs. 46.21 ± 10.89, min, *p* < 0.001) in acromegaly patients compared with the controls. DNMBD and CNMBD decreased in patients with decreasing body fat percentage and increasing growth hormone (GH) level, insulin-like growth factor 1 (IGF-1) level, and GH and IGF-1 burden. The onset time increased with increasing IGF-1 level and GH and IGF-1 burden. Taken together, a unique NMB status was identified in acromegaly patients with the following characteristics: prolonged onset time and shortened DNMBD and CNMBD. Changes in the levels and burdens of GH and IGF-1 and body composition were linearly correlated with intraoperative NMB in acromegaly patients.

## 1. Introduction

Acromegaly is a rare neuroendocrine disease in the field of neurosurgery with an incidence of five per one million per year and a prevalence of 60 per one million [1]. Excess growth hormone (GH) and insulin-like growth factor 1 (IGF-1) in patients with acromegaly can influence the whole body to cause bone and soft tissue hypertrophy [2]. Peripheral organ enlargement, facial changes, myocardial hypertrophy, and airway stenosis are also common presentations in these patients [3–5]. Excess GH and IGF-1 can also result in changes in the body composition of patients with acromegaly. These changes include abnormal adipose tissue distribution and elevated lean body mass, skeletal muscle mass, total body water, and extracellular water [6, 7]. Because more than 95% of acromegaly cases are caused by a GH-secreting pituitary adenoma, transsphenoidal pituitary adenoma resection under general anesthesia is the first-line therapy for these cases [5]. Proper control of general anesthesia, including sedation, analgesia, and neuromuscular blockade (NMB), is a prerequisite for successful surgery. Retention of NMB agents can cause delayed anesthesia recovery and weak cough reflex, which is associated with residual curarization, increasing the risk for pulmonary atelectasis and pneumonia [8]. However, insufficient NMB increases throat muscle tension and enhances the difficulty of tracheal intubation [9–11].

In clinical practice, it is difficult to expose the vocal cords of patients with acromegaly. In our previous study, we found that the tracheal intubation time is prolonged and the average number of attempts before successful intubation is increased in acromegaly patients [12]. These conditions could increase the difficulty of surgery or lead to a failed operation. Although these endotracheal intubation difficulties are related to upper airway stenosis and a thickened pharyngeal wall, a sufficient neuromuscular blockade is required to optimize the conditions for endotracheal intubation. During surgery, deep NMB ameliorates surgical conditions [13]. According to the literature, the characteristics of NMB have been well studied in obese and elderly patients [9, 10, 14]. The NMB effects in these groups of patients differ from those in the general population, and changes in body composition and drug metabolism are the influencing factors. However, whether and to what extent the NMB characteristics are altered in patients with acromegaly remains unclear, and the correlations with hormone levels and patients' body composition are unknown.

Therefore, this study aimed to identify the onset time of NMB drugs and the deep neuromuscular blockade duration (DNMBD) and clinical neuromuscular blockade duration (CNMBD) of patients with acromegaly during surgery and anesthesia and to analyze the correlations between NMB, pituitary hormones, and body composition.

## 2. Materials and Methods

### 2.1. Subjects

This is a single-center, prospective, case-control study. The experimental group included consecutively enrolled patients with acromegaly. The inclusion criteria were as follows: (1) patients presenting with acromegaly-related manifestations; (2) patients meeting the endocrinological diagnostic standard [2]; (3) patients with pituitary adenoma on contrast-enhanced magnetic resonance imaging (MRI); (4) patients who had never received related pituitary surgery, irradiation, or medical treatment; (5) patients with other pituitary-related hormones within normal reference levels. The exclusion criteria were as follows: (1) patients with in-body metal; (2) patients with previous neuromuscular diseases; (3) patients with hypokalemia and patients taking steroids, anticonvulsants, amides, or other drugs; (4) patients with primary heart, liver, biliary tract, or kidney diseases. Patients with nonfunctioning pituitary adenomas, matched according to sex and age with the patients in the experimental group, were enrolled as the control group. The inclusion criteria were as follows: (1) patients with all pituitary-related hormones within normal reference levels; (2) patients with pituitary adenoma on MRI; (3) patients who have never received pituitary surgery, irradiation, or medical treatment. The exclusion criteria were the same as those for the experimental group.

Informed consent was obtained from each patient before enrollment. This study was approved by the Ethics Committee of Peking Union Medical College Hospital at the Chinese Academy of Medical Sciences and Peking Union Medical College.

### 2.2. Study Design

Necessary preoperative tests were performed after the admission of patients to assist in definitive diagnosis and to prepare for the operation. The hormones tested included prolactin, thyroid-stimulating hormone, thyroxine, triiodothyronine, free thyroxine, free triiodothyronine, adrenocorticotropic hormone, cortisol, progestin, estrogen 2, testosterone, follicle-stimulating hormone, and luteinizing hormone. Body compositions were analyzed, and indexes included the lean body mass, total muscle mass, skeletal muscle mass, and percentage of body fat. Information on the sex, age, height (accurate to 0.1 cm), weight (accurate to 0.1 kg), body mass index, disease history, hormone levels, and body composition was recorded in detail for each patient.

### 2.3. Hormone Assays and Body Composition Analysis

Serum hormones were collected at 6 a.m. after the patients had fasted for at least eight hours. The hormone measurement method was consistent with that of our previous study [12]. The disease duration indicated the duration from the onset of the disease to a definite diagnosis. GH burden was defined as the product of random GH and disease duration, and IGF-1 burden was defined as the product of IGF-1 and disease duration.

The body composition of the patients was determined with a body composition analyzer (seca mBCA515, Germany). Patients were required to walk into the measurement room without any metallic belongings in the morning after an eight-hour fasting period. After their palms and feet were cleaned, the patients sat down and relaxed for 5 min, and the measurement was commenced. The patients stood straight with their bare feet in contact with the electrodes on the measurement table and their hands in contact with the electrodes on the handrail. The internal software of the body composition analyzer calculated the results within 70 seconds. Detailed information on this process can be referred to in our previously published article [15].

### 2.4. Neuromuscular Monitoring

In the operating room, a 16-G peripheral venous line was connected to the patient's left arm, while the right arm was immobilized with a cuff. We performed NMB in accordance with specific guidelines [16]. NMB was assessed using the train-of-four (TOF) technique with the TOF-Watch® SX (Organon, Oss, Netherlands). Four consecutive stimuli were delivered along the ulnar nerve, and muscle contraction was measured to evaluate the effects. Four equal muscle contractions indicated the absence of NMB, whereas decreases in the number and height of contractions indicated the degree of blockade.

### 2.5. General Anesthesia

Anesthesia was performed throughout the procedure by the same anesthetist (Zhang Y). Anesthesia was induced with midazolam (0.2 mg/kg), sufentanil (0.15 *μ*g/kg), and propofol (6 *μ*g/ml) using a target-controlled infusion (TCI) system. Once the patient became unconscious, a 50-Hz tetanus stimulation was applied for 5 sec. After the baseline of the equipment was recovered and stabilized (<5% variation in at least 2 min), supramaximal stimulation and calibration were ensured. The patients were ventilated via a mask, while the NMB monitoring equipment was calibrated. Rocuronium (0.6 mg/kg) was then administered within 5 sec through a rapidly flowing intravascular line and flushed with 5 ml saline. The TOF stimulation was set to repeat every 15 sec. Tracheal intubation was performed when the TOF ratio decreased to zero. The TOF ratio was defined as the amplitude of the fourth response to stimuli divided by the amplitude of the first response [16, 17]. Each time the first response to TOF (T1) recovered to 25%, 0.1 mg/kg rocuronium was administered. After the surgery, tracheal extubation was performed when the patients recovered total consciousness and the TOF ratio exceeded 0.9.

The onset time and DNMBD and CNMBD, the most commonly used indexes to evaluate the extent of NMB [18–20], were recorded during anesthesia. The onset time was defined as the time from rocuronium administration to the time when the TOF ratio decreased to zero. Difference of more than ten seconds of the onset time is regarded to be clinically significant. DNMBD was defined as the recovery time from the TOF ratio of zero to the value at T1 after the first dose of rocuronium. CNMBD was defined as the time when T1 recovered to 25% of its control value.

### 2.6. Statistical Analysis

We performed all statistical analyses with SPSS software (version 17.0, IBM, USA) and constructed scatter plots with GraphPad software (Prism 5.01, GraphPad Software Inc. USA). Continuous quantitative data are shown as the means ± standard deviation, and discrete data are shown as numbers and percentages. Levene's test was used to evaluate the distribution of the quantitative data; data with normal distributions were assessed using *t*-tests, whereas variables lacking normal distributions were assessed using the Mann–Whitney U test. Discrete data were compared using the *χ*^2^ test. Linear regressions were performed for the NMB indexes with body composition parameters and pituitary hormone levels. The results are presented as scatter plots and linear regression equations. Statistical significance was defined at *p* < 0.05.

## 3. Results

### 3.1. Subjects and Clinical Basis

Twenty patients with acromegaly (11 males and 9 females) and 17 patients with nonfunctioning pituitary adenoma (5 males and 12 females) were enrolled into this study. Clinical information, body composition and hormone levels in the two groups are summarized in Table 1. No significant differences in sex, age, height, weight, body mass index, and disease history were identified between the two groups. The largest tumor diameter (mm, 18.20 ± 9.41 vs. 24.97 ± 13.34) and the waist to hip ratio (0.88 ± 0.06 vs. 0.91 ± 0.06) were similar in the two groups. The body composition and hormone levels of the patients with acromegaly were significantly different from those of the controls. The lean body mass, total muscle mass, and skeletal muscle mass were significantly higher, whereas the percentage of body fat was markedly lower. Both random GH levels and IGF-1 levels were elevated in patients with acromegaly. In addition, the GH burden and the IGF-1 burden were also markedly increased. No differences were observed among the other pituitary-related hormones.

### 3.2. Characteristics of Neuromuscular Blockade in Acromegaly

Data collection was performed during the surgery, and the data were transferred from the TOF-Watch SX Monitor Software into Microsoft Excel immediately after surgery. The characteristics of NMB in the two groups are presented in Table 2. Although the NMB agent was administered based on the patient's total body weight, its effects were not satisfactory in patients with acromegaly. Compared with controls, in patients with acromegaly, the average onset time was prolonged by 35 seconds (47%) (*p*=0.017), whereas the average DNMBD was shortened by 13 min (37%, *p* < 0.001), and the average CNMBD was shortened by 13 min (28%, *p* < 0.001).

### 3.3. Factors Correlated with Neuromuscular Blockade

**Figure 1:**
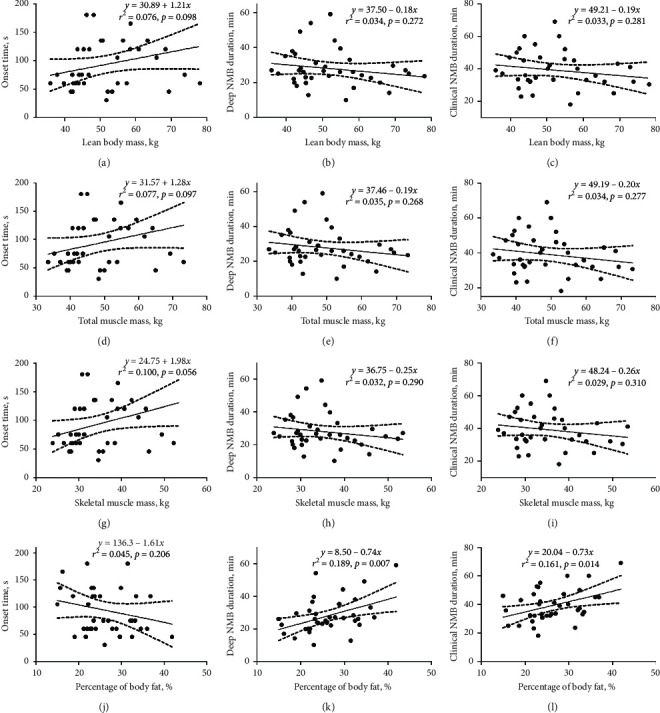
Scatter plots presenting correlations between body composition parameters and neuromuscular blockade (NMB) indexes. The body composition parameters include lean body mass, total muscle mass, skeletal muscle mass, and percentage of body fat. In each scatter plot, the solid straight line is the best fit line representing the linear regression result. The dashed curves on both sides of the best fit line indicate the 95% confidence interval. The linear regression equation, *r*^2^, and (*p*) value are labeled on the top right corner of the scatter plot.

**Figure 2:**
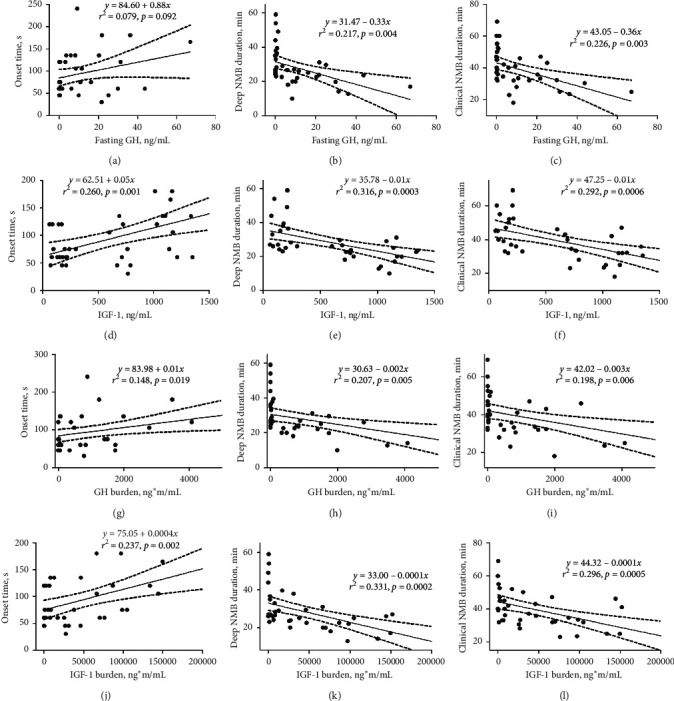
Scatter plots representing correlations between hormone indexes and neuromuscular blockade (NMB) indexes. The hormone indexes included random GH, IGF-1, GH burden, and IGF-1 burden. The GH burden was defined as the product of random GH and disease duration, and the IGF-1 burden was defined as the product of IGF-1 and disease duration. In each scatter plot, the solid straight line is the best fit line representing the linear regression result. The dashed curves on both sides of the best fit line indicate the 95% confidence interval. The linear regression equation, *r*^2^, and (*p*) value are labeled on the top right corner of the scatter plot.

According to the results of baseline comparisons, we set the body composition indexes (lean body mass, total muscle mass, skeletal muscle mass, and percentage of body fat) and hormone-related indexes (GH, IGF-1, GH burden, and IGF-1 burden) as the abscissa and the onset time, DNMBD, and CNMBD as the ordinates to construct scatter plots and perform linear regression analyses. The results are presented in Figures 1 and 2. The *r*^2^ values for percentage of body fat, GH, IGF-1, GH burden, and IGF-1 burden were relatively high, indicating a higher potential applicability among all the indexes in clinical practice. With the increase in IGF-1, GH burden, and IGF-1 burden, the onset time of rocuronium was prolonged, and the DNMBD and CNMBD were shortened. The DNMBD and CNMBD were shortened with increased GH and decreased percentage of body fat. Although the onset time exhibited an increasing trend with increased GH and decreased percentage of body fat, the slope was not significantly different from zero. Likewise, with increased lean body mass, total muscle mass, and skeletal muscle mass, the onset time increased, and the DNMBD and CNMBD decreased, although the trends were not significant.

In clinical practice, the standard values for onset time and CNMBD of rocuronium are 90 seconds and 40 min, respectively [21–23]. We stratified the acromegaly patients into subgroups according to these standards. Clinical information, body compositions, and hormone levels were compared among the subgroups, but no remarkable differences were detected.

## 4. Discussion

In this study, we demonstrated that the onset time of rocuronium was prolonged by 47%, while the DNMBD and CNMBD were shortened by 37% and 28%, respectively, during anesthesia in patients with acromegaly compared with controls. The percentage of body fat was reduced, and the lean body mass, total muscle mass, and skeletal muscle mass were elevated in acromegaly patients. With a decreasing percentage of body fat and increasing GH, IGF-1, GH burden, and IGF-1 burden, the DNMBD and CNMBD both significantly decreased in a linear manner in acromegaly patients. Additionally, with increasing IGF-1, GH burden, and IGF-1 burden, the onset time was linearly prolonged.

Fluctuations in NMB can increase perioperative complications, including muscle weakness and unsatisfactory cough reflex, leading to a higher risk of regurgitation, aspiration, pulmonary atelectasis, and pneumonia [24, 25]. However, studies on insufficient NMB are lacking. Our study demonstrated that the onset time of rocuronium was prolonged, and the effective duration was shortened, indicating insufficient NMB in acromegaly patients.

The efficacy of NMB agents has been reported to be correlated with body composition, age, metabolism status, cardiac output, and liver, biliary tract, or kidney diseases [26–29]. Thus, we excluded patients with heart, liver, biliary tract, or kidney diseases from our study and matched the control group by age. The ammonium moiety in rocuronium makes it hydrophilic and enables it to be metabolized mostly in the fat-free mass [9]. In obese individuals, the percentage of body fat is elevated, whereas the fat-free mass is reduced. Therefore, the “distribution volume” of rocuronium is decreased in these individuals [28, 29]. In these patients, the blood concentration of rocuronium can increase rapidly, and the time for it to decrease from a relatively higher level to the threshold value can be prolonged [10, 30].

Contrary to the changes in body composition in obesity, the percentage of body fat in patients with acromegaly is lower than that in the controls. This finding may be because excess GH, as a lipolytic hormone, can lead to reduced body fat and adipose tissue distribution [6, 7, 31]. The distribution volume theory can explain the relationship between the extent of NMB and changes in body composition in acromegaly. In these patients, the distribution volume is elevated because of the reduced percentage of body fat. Thus, after rocuronium administration, its concentration in the blood likely increases slowly per unit volume, thereby prolonging the time before the onset of NMB. Therefore, despite the identical total dosage of rocuronium, its blood concentration per unit volume is lower, and the effective duration is shorter under the same metabolic conditions.

Elevated GH and IGF-1 levels commonly noted in patients with acromegaly and are also the basis of many differences between acromegaly and other diseases. Our study, for the first time, showed that GH, IGF-1, GH burden, and IGF-1 burden are important factors influencing the pharmacokinetics and pharmacodynamics of NMB agents. Studies investigating the correlations between hormone levels and clinical NMB are lacking, and the associated mechanisms remain unknown. In previous studies, the basal metabolic rate has been reported to be elevated in patients with acromegaly or individuals receiving GH replacement therapy [32]. In these patients, oxygen consumption is increased due to accelerated anabolism, secondary hyperinsulinemia leading to enhanced glucose metabolism, and increased lean body mass promoting the activity of Na+/K + -ATPase. Thus, the elevated metabolism in patients with acromegaly accelerates the synthesis, degradation, and biotransformation of substrates [33–35]. It can be speculated that when rocuronium is administered to patients with acromegaly, the drug is metabolized faster, and its blood concentration is reduced, leading to the prolonged onset time and the shortened effective duration of NMB during surgery.

This study has some limitations. Because acromegaly is a rare neuroendocrine disease with an incidence of five cases per million per year [11], patient enrollment was difficult, and the sample size in this study was small, which could lead to a low reliability of the statistical analysis results. Future multicenter studies may provide a larger sample size and more reliable and precise results. Moreover, we defined the disease duration as the period from the onset of the acromegaly-associated symptoms to a clinical diagnosis of acromegaly, but there would always be inevitable inaccuracy. Furthermore, although the bioimpedance parameters used in this study are widely accepted and used, their accuracy are not as high as the whole-body MRI and dual energy X-ray absorptiometry in evaluating the body composition.

In conclusion, NMB is unsatisfactory in patients with acromegaly, who present with prolonged onset time and shortened DNMBD and CNMBD. With increasing GH, IGF-1, GH burden, and IGF-1 burden and decreasing percentage of body fat, the onset time is prolonged, and the DNMBD and CNMBD are shortened linearly. Thus, hormone levels, hormonal burdens, and body composition can help predict the effects of NMB before surgery. Further mechanistic studies on the impact of hormone levels and body composition on NMB agents are warranted.

## Figures and Tables

**Table 1:** Clinical characteristics, body composition, and hormone levels of patients with acromegaly and controls.

	Patients with acromegaly (*n* = 20)	Controls (*n* = 17)	*p*
Male, *n* (%)	11 (55.00)	5 (29.41)	0.185
Age, years	42.15 ± 9.78	48.18 ± 13.12	0.119
Height, cm	168.75 ± 7.69	164.65 ± 6.88	0.099
Weight, kg	73.60 ± 14.72	67.81 ± 11.64	0.199
Body mass index	25.76 ± 3.62	24.98 ± 3.86	0.583
Hypertension, *n* (%)	5 (25.00)	3 (17.65)	0.701
Diabetes mellitus, *n* (%)	4 (20.00)	2 (11.76)	0.667
Lean body mass, kg	56.46 ± 11.59	47.31 ± 7.52	0.007
Total muscle mass, kg	52.86 ± 10.97	44.19 ± 7.12	0.007
Skeletal muscle mass, kg	37.96 ± 8.23	31.58 ± 5.08	0.007
Body fat percentage, %	23.24 ± 5.04	29.86 ± 5.77	0.001
GH nadir, ng/mL	11.69 ± 7.53	NA	NA
Random GH, ng/mL	19.59 ± 16.04	0.37 ± 0.39	<0.001
IGF-1, ng/mL	1005.00 ± 274.77	160.76 ± 66.29	<0.001
GH burden, ng^*∗*^m/mL	1694.72 ± 1977.98	13.58 ± 22.95	0.001
IGF-1 burden, ng^*∗*^m/mL	85427.50 ± 62004.96	7483.82 ± 11191.55	<0.001

GH: growth hormone; IGF-1: insulin-like growth factor 1.

**Table 2:** Muscle relaxation parameters in patients with acromegaly and controls.

	Patients with acromegaly (*n* = 20)	Controls (*n* = 17)	*p*
OT, seconds	110.25 ± 54.90	75.00 ± 27.56	0.017
CNMBD, minutes	21.99 ± 5.67	34.96 ± 11.04	<0.001
DNMBD, minutes	33.26 ± 8.09	46.21 ± 10.89	<0.001

OT: onset time; CNMBD: clinical neuromuscular blockade duration; DNMBD deep neuromuscular blockade duration.

## Data Availability

All data analyzed in this study are available upon reasonable request by contacting the corresponding author.

## References

[1] Kreitschmann-Andermahr I., Siegel S., Kleist B. (2016). Diagnosis and management of acromegaly: the patient’s perspective. *Pituitary*.

[2] Katznelson L., Laws E. R., Melmed S. (2014). Acromegaly: an endocrine society clinical practice guideline. *The Journal of Clinical Endocrinology & Metabolism*.

[3] Melmed S., Casanueva F. F., Klibanski A. (2013). A consensus on the diagnosis and treatment of acromegaly complications. *Pituitary*.

[4] Dineen R., Stewart P. M., Sherlock M. (2017). Acromegaly–diagnosis and clinical management. *QJM*.

[5] Vilar L., Vilar C. F., Lyra R., Lyra R., Naves L. A. (2017). Acromegaly: clinical features at diagnosis. *Pituitary*.

[6] Birzniece V., Nelson A. E., Ho K. K. Y. (2011). Growth hormone and physical performance. *Trends in Endocrinology & Metabolism*.

[7] Reyes-Vidal C. M., Mojahed H., Shen W. (2015). Adipose tissue redistribution and ectopic lipid deposition in active acromegaly and effects of surgical treatment. *The Journal of Clinical Endocrinology & Metabolism*.

[8] Berg H., Viby-mogensen J., Roed J. (1997). Residual neuromuscular block is a risk factor for postoperative pulmonary complications A prospective, randomised, and blinded study of postoperative pulmonary complications after atracurium, vecuronium and pancuronium. *Acta Anaesthesiologica Scandinavica*.

[9] Ingrande J., Lemmens H. J. M. (2013). Anesthetic pharmacology and the morbidly obese patient. *Current Anesthesiology Reports*.

[10] Van Kralingen S., Van De Garde E. M. W., Knibbe C. A. J. (2011). Comparative evaluation of atracurium dosed on ideal body weight vs. total body weight in morbidly obese patients. *British Journal of Clinical Pharmacology*.

[11] Grayling M., Sweeney B. P. (2007). Recovery from neuromuscular blockade: a survey of practice. *Anaesthesia*.

[12] Zhang Y., Guo X., Pei L., Zhang Z., Tan G., Xing B. (2017). High levels of IGF-1 predict difficult intubation of patients with acromegaly. *Endocrine*.

[13] Blobner M., Frick C. G., Feussner R. B. (2015). Neuromuscular blockade improves surgical conditions (NISCO). *Surgical Endoscopy*.

[14] Claudius C., Karacan H., Viby-Mogensen J. (2007). Prolonged residual paralysis after a single intubating dose of rocuronium. *British Journal of Anaesthesia*.

[15] Guo X., Gao L., Shi X. (2018). Pre- and postoperative body composition and metabolic characteristics in patients with acromegaly: a prospective study. *Internation Journal of Endocrinology*.

[16] Fuchs-Buder T., Claudius C., Skovgaard L. T., Eriksson L. I., Mirakhur R. K., Viby-Mogensen J. (2007). Good clinical research practice in pharmacodynamic studies of neuromuscular blocking agents II: the Stockholm revision. *Acta Anaesthesiologica Scandinavica*.

[17] Engbaek J., Ostergaard D., Viby-Mogensen J., Skovgaard L. T. (1989). Clinical recovery and train-of-four ratio measured mechanically and electromyographically following atracurium. *Anesthesiology*.

[18] Groudine S. B., Soto R., Lien C., Drover D., Roberts K. (2007). A randomized, dose-finding, phase II study of the selective relaxant binding drug, Sugammadex, capable of safely reversing profound rocuronium-induced neuromuscular block. *Anesthesia & Analgesia*.

[19] Duvaldestin P., Kuizenga K., Saldien V. (2010). A randomized, dose-response study of sugammadex given for the reversal of deep rocuronium- or vecuronium-induced neuromuscular blockade under sevoflurane anesthesia. *Anesthesia & Analgesia*.

[20] Sparr H. J., Vermeyen K. M., Beaufort A. M. (2007). Early reversal of profound rocuronium-induced meeting abstracts by sugammadex in a randomized multicenter study. *Anesthesiology*.

[21] Naguib M. (1994). Neuromuscular effects of rocuronium bromide and mivacurium chloride administered alone and in combination. *Anesthesiology*.

[22] Magorian T., Flannery K. B., Miller R. D. (1993). Comparison of rocuronium, succinylcholine, and vecuronium for rapid-sequence induction of anesthesia in adult patients. *Anesthesiology*.

[23] Meistelman C., Plaud B., Donati F. (1992). Rocuronium (ORG 9426) neuromuscular blockade at the adductor muscles of the larynx and adductor pollicis in humans. *Canadian Journal of Anaesthesia*.

[24] Arbous M. S., Meursing A. E. E., Van Kleef J. W. (2005). Impact of anesthesia management characteristics on severe morbidity and mortality. *Anesthesiology*.

[25] Murphy G. S., Szokol J. W., Franklin M., Marymont J. H., Avram M. J., Vender J. S. (2004). Postanesthesia care unit recovery times and neuromuscular blocking drugs: a prospective study of orthopedic surgical patients randomized to receive pancuronium or rocuronium. *Anesthesia & Analgesia*.

[26] van Miert M. M., Eastwood N. B., Boyd A. H., Parker C. J. R., Hunter J. M. (1997). The pharmacokinetics and pharmacodynamics of rocuronium in patients with hepatic cirrhosis. *British Journal of Clinical Pharmacology*.

[27] Leykin Y., Pellis T., Lucca M., Lomangino G., Marzano B., Gullo A. (2004). The pharmacodynamic effects of rocuronium when dosed according to real body weight or ideal body weight in morbidly obese patients. *Anesthesia & Analgesia*.

[28] Kendrick J. G., Carr R. R., Ensom M. H. (2010). Pharmacokinetics and drug dosing in obese children. *The Journal of Pediatric Pharmacology and Therapeutics*.

[29] Casati A., Putzu M. (2005). Anesthesia in the obese patient: pharmacokinetic considerations. *Journal of Clinical Anesthesia*.

[30] Patanwala A. E., Stahle S. A., Sakles J. C., Erstad B. L. (2011). Comparison of succinylcholine and rocuronium for first-attempt intubation success in the emergency department. *Academic Emergency Medicine*.

[31] Katznelson L. (2009). Alterations in body composition in acromegaly. *Pituitary*.

[32] Salomon F., Cuneo R. C., Hesp R., Morris J. F., Poston L., Sönksen P. H. (1992). Basal metabolic rate in adults with growth hormone deficiency and in patients with acromegaly: relationship with lean body mass, plasma insulin level and leucocyte sodium pump activity. *Clinical Science*.

[33] Ng L. L., Evans D. J. (1987). Leucocyte sodium transport in acromegaly. *Clinical Endocrinology*.

[34] Jørgensen J. O., Møller L., Krag M., Billestrup N., Christiansen J. S. (2007). Effects of growth hormone on glucose and fat metabolism in human subjects. *Endocrinology and Metabolism Clinics of North America*.

[35] Hammarqvist F., Wennstrom I., Wernerman J. (2010). Effects of growth hormone and insulin-like growth factor-1 on postoperative muscle and substrate metabolism. *Journal of Nutrition and Metabolism*.

